# Scaffold-free tissue engineering for injured joint surface restoration

**DOI:** 10.1186/s40634-017-0118-0

**Published:** 2018-01-05

**Authors:** Kazunori Shimomura, Wataru Ando, Hiromichi Fujie, David A. Hart, Hideki Yoshikawa, Norimasa Nakamura

**Affiliations:** 1 Medicine for Sports and Performing Arts, Department of Health and Sport Sciences, Osaka University Graduate School of Medicine, 2-2 Yamadaoka, Suita City, Osaka 565-0871 Japan; 2 Department of Orthopaedic Surgery, Osaka University Graduate School of Medicine, 2-2 Yamadaoka, Suita City, Osaka 565-0871 Japan; 3 Division of Human Mechatronics Systems, Faculty of System Design, Tokyo Metropolitan University, 6-6 Asahigaoka, Hino City, Tokyo 191-0065 Japan; 4 McCaig Institute for Bone & Joint Health, University of Calgary, 3330 Hospital Drive Northwest, Calgary, AB T2N 4N1 Canada; 5 Institute for Medical Science in Sports, Osaka Health Science University, 1-9-27 Tenma, Kita-ku, Osaka City, Osaka 530-0043 Japan; 6 Center for Advanced Medical Engineering and Informatics, Osaka University, 2-2 Yamadaoka, Suita City, Osaka 565-0871 Japan

**Keywords:** Scaffold-free, Mesenchymal stem cell, Cartilage repair

## Abstract

Articular cartilage does not heal spontaneously due to its limited healing capacity, and thus effective treatments for cartilage injuries has remained challenging. Since the first report by Brittberg et al. in 1994, autologous chondrocyte implantation (ACI) has been introduced into the clinic. Recently, as an alternative for chondrocyte-based therapy, mesenchymal stem cell (MSC)-based therapy has received considerable research attention because of the relative ease in handling for tissue harvest, and subsequent cell expansion and differentiation. In this review, we discuss the latest developments regarding stem cell-based therapies for cartilage repair, with special focus on recent scaffold-free approaches.

## Background

Dealing with articular cartilage injuries is quite common in the fields of orthopaedic surgery and musculoskeletal medicine. Due to its avascular and aneural surroundings, as well as its relatively unique matrix organization, articular cartilage does not heal in response to the currently available treatments, and subsequently, the injury may lead to the development of osteoarthritis. Therefore, a variety of approaches have been tested to improve cartilage healing over the past few decades (Huang et al. 2016; Vavken and Samartzis 2010; Krych et al. 2016).

Since the first report of autologous chondrocyte implantation (ACI) was published in 1994 by Brittberg et al. (Brittberg et al. 1994), chondrocyte-based therapies have been extensively studied, some of which have been successfully introduced into the clinic (Niemeyer et al. 2014; Goyal et al. 2013; Steinwachs and Kreuz 2007). On the other hand, these procedures have some limitations including the sacrifice of undamaged cartilage within the same joint, as well as potential cellular alterations associated with the in vitro expansion of the chondrocytes. Furthermore, due to the degenerative changes in cartilage that can accompany aging, the availability of cells may be limited in elderly individuals, both quantitatively and qualitatively (Hickery et al. 2003).

To overcome such potential problems, stem cell-based therapies have been the focus of attention to facilitate regenerative tissue repair. Mesenchymal stem cells (MSCs) have the capability to differentiate into a variety of connective tissue cells including bone, cartilage, tendon, muscle, and adipose tissue (Pittenger et al. 1999). These cells can be isolated from various tissues such as bone marrow, skeletal muscle, synovial membrane, adipose tissue, and umbilical cord blood (Pittenger et al. 1999; Jankowski et al. 2002; De Bari et al. 2001; Sakaguchi et al. 2005; Wickham et al. 2003; Lee et al. 2004), as well as synovial fluid (Ando et al. 2014). MSCs isolated from synovium may be well suited for cell-based therapies for cartilage because of the relative ease of harvest and their strong capability for chondrogenic differentiation (De Bari et al. 2001). Among mesenchymal tissue-derived cells, synovium-derived cells are reported to exhibit the greatest chondrogenic potential (Sakaguchi et al. 2005). As other options for a cell source, allogeneic synovial or bone marrow MSCs (Shimomura et al. 2010; Dashtdar et al. 2011) or induced pluripotent stem (iPS) cells (Takahashi and Yamanaka 2006; Tsumaki et al. 2015) have been assessed. However, not much conclusive evidence using these cells has yet been forth coming in terms of preclinical and clinical safety, and thus further studies with such cells are likely still required.

With the advancement of cell-based technologies, many of the recent tissue engineering approaches have taken advantage of scaffolds made of either synthetic (Vunjak-Novakovic et al. 1999; Andriano et al. 1999; Guo et al. 1989; Masuda et al. 2003) or natural polymers (Lee et al. 2001; Homminga et al. 1993; Brun et al. 1999; Lahiji et al. 2000; Shimomura et al. 2014) as the supporting biomaterials, providing an appropriate three-dimensional (3D) environment for cells to optimize their proliferation and chondrogenic differentiation (De Bari et al. 2004), and effectively delivering cells into cartilage defects (Bright and Hambly 2014; Gobbi et al. 2009). This technique is robust and easy for surgeons to handle, and was reported to significantly improve the healing of cartilage defects. Interestingly, a recent systematic review indicated that the scaffold-based ACI methodology using collagen scaffold (Matrix-induced ACI; MACI) provided better clinical results than did the use of chondrocytes alone, but the evidence is still not strong (Goyal et al. 2013). On the other hand, there are still several issues associated with the long-term safety and efficacy of these materials. Synthetic polymers may have potential problems regarding retention and degradation in situ (Daniels et al. 1994; van der Elst et al. 1999). Biological materials potentially carry the risk of transmission of infectious agents and initiating immunological reactions (Yang et al. 2004; Martin et al. 2005). Taken together, and in order to minimize unknown risk, such materials may ideally be excluded throughout the treatment procedure. However, there have been no direct comparative studies of scaffold-based and scaffold-free approaches reported and thus, it is still unknown which approache(s) would provide better long-term outcomes. In spite of this limitations, a scaffold-free cell delivery system may be an excellent alternative due to its simplicity in development and ease of subsequent implantation. In this review, we discuss the latest developments regarding cell-based therapies for cartilage repair, focusing specifically on recent scaffold-free approaches, in the first section. Subsequently, we focus the second half of the review on our experiences regarding development of a unique MSC-based scaffold-free approach, taking a bench to bedside for clinical application approach.

## Review

### Recent studies of scaffold-free cell-based therapies for cartilage repair

We searched MEDLINE for reports published in English up to November, 2017, using the terms “scaffold free”, “scaffold less” and “cartilage repair”, with the exception of studies using simple intra-articular cell injection. Additionally, we collected related researches at second hand as far as possible. We identified 48 publications, of which 26 were related to in-vitro experiments, 21 reported in-vivo tests, and one was a clinical study as listed in Table 1. Also, three review articles were identified (Shimomura et al. 2015; Yasui et al. 2016; DuRaine et al. 2015).

**Table 1:** Summary of scaffold-free cell-based therapies for cartilage repair

Authors	Journal	Year	Experimental Model	Scaffold-free Technique	Shape	Cell Source	Matrix	Amount of Cells	Defect Size (in vivo)	Graft fixation (in vivo)
Adkisson HD	Clin Orthop Relat Res	2001	in vitro	High-density in Monolayer	Disc	Chondrocyte	Catilage-like	2 × 10^5^/cm^2^ per plate	N/A	N/A
Mainil-Varlet P, et al.	Osteoarthritis Cartilage	2001	Minipig	Bioreactor	Spheroid	Chondrocyte	Catilage-like	2 × 10^7^	4 mm diameter(Full-thickness cartilage defect)	Press-fit
Anderer U, et al.	J Bone Miner Res	2002	in vitro	Self Aggregating	Spheroid	Chondrocyte	Catilage-like	2 × 10^5^	N/A	N/A
Masuda K, et al.	J Orthop Res	2003	in vitro	Alginate Recovered Chondrocyte Method	Sheet	Chondrocyte	Catilage-like	4 × 10^6^per mL	N/A	N/A
Lu Y, et al.	J Knee Surg	2005	Sheep	High-density in Monolayer	Disc	Chondrocyte	Catilage-like	2 × 10^5^/cm^2^ per plate	5.5 mm diameter 400–500 μm depth	Suture
Stoddart MJ, et al.	J Cell Mol Med	2006	in vitro	Cell Pellet After Culture in Alginate	Disc	Chondrocyte	Catilage-like	4 × 10^6^per mL	N/A	N/A
Stoddart MJ, et al.	Biotechnol Bioeng	2006	in vitro	Cell Pellet After Culture in Alginate	Spheroid	Chondrocyte	Catilage-like	4 × 10^6^per mL	N/A	N/A
Kaneshiro N, et al.	Biochem Biophys Res Commun	2006	in vitro	Layered Chondrocyte Sheet	Sheet	Chondrocyte	Catilage-like	1 × 10^4^per cm^2^	N/A	N/A
Hu JC, et al.	Tissue Eng	2006	in vitro	High-density in Monolayer	Disc	Chondrocyte	Catilage-like	5.5 × 10^6^	N/A	N/A
Park K, et al.	Artificial Organs	2006	Pig	High-density in Monolayer	Disc	Chondrocyte	Catilage-like	1.9 × 10^5^/cm^2^per plate	6 mm diameter 3–4 mm depth	Bovine collagen gel
Brehm W, et al.	Osteoarthritis Cartilage	2006	Goat	Bioreactor	Disc	Chondrocyte	Catilage-like	2 × 10^7^	6 mm diameter 0.8 mm depth	Periosteal falp, PRP, Fibrin
Hayes AJ, et al.	J Histochem Cytochem	2007	in vitro	High-density in Monolayer	Sheet	Chondrocyte	Catilage-like	6 × 10^6^	N/A	N/A
^a^Ando W, et al.	Biomaterials	2007	Pig	High-density in Monolayer	Sheet	Synovial MSC	Collagen I, III-rich	4 × 10^5^/cm^2^per plate	8.5 mm diameter2 mm depth	Nether glue nor suture
Murdoch AD, et al.	Stem Cells	2007	in vitro	Transwell Culture	Disc	Bone Marrow MSC	Catilage-like	5 × 10^5^	N/A	N/A
^a^Ando W, et al.	Tissue Eng Part A	2008	in vitro	High-density in Monolayer	Sheet	Synovial MSC	Catilage-like	4 × 10^5^/cm^2^per plate	N/A	N/A
Ofek G, et al.	ProS One	2008	in vitro	High-density in Monolayer	Spheroid	Chondrocyte	Catilage-like	5.5 × 10^6^	N/A	N/A
Nagai T, et al.	Tissue Eng Part A	2008	Rabbit	High-density in Monolayer	Disc	Chondrocyte	Catilage-like	6 × 10^6^	5 mm diameter3 mm depth	Nether glue nor suture
Jubel A, et al.	Am J Sports Med	2008	Sheep	Cell Pellet After Culture in Alginate	Disc	Chondrocyte	Catilage-like	4 × 10^6^ per mL	4 mm diameter(Full-thickness cartilage defect)	Periosteal flap
Schubert T, et al.	Int J Mol Med	2009	Mouse	Self Aggregating	Spheroid	Chondrocyte	Catilage-like	2 × 10^5^	5 mm diameter3-4 mm depth(Human cartilageco-implant model)	Nether glue nor suture
Lewis PB, et al.	J Knee Surg	2009	Goat	High-density in Monolayer	Disc	Chondrocyte	Catilage-like	2 × 10^5^/cm^2^per plate	4 or 6 mm diameter	Fibrin glue
^a^Shimomura K, et al.	Biomaterials	2010	Pig	High-density in Monolayer	Sheet	Synovial MSC	Collagen I, III-rich	4 × 10^5^/cm^2^per plate	8.5 mm diameter2 mm depth	Nether glue nor suture
Kraft JJ, et al.	Cartilage	2011	in vitro	Self Aggregating	Disc	Chondrocyte	Catilage-like	2 × 10^7^per mL	N/A	N/A
Zhang B, et al.	Tissue Eng Part C	2011	in vitro	Self Aggregating	Round or Oval	Bone MarrowMSC	Catilage-like	2.5 × 10^7^per mL	N/A	N/A
Maeda S, et al.	J Biosci Bioeng	2011	in vitro	Culture on Membrane (polyethylene terephthalate)	Disc	Bone MarrowMSC	Catilage-like	3.1 × 10^6^	N/A	N/A
Cheuk YC, et al.	J Orthop Res	2011	Rabbit	Pellet Culture	Spheroid	AllogenicChondrocyte	Not assessed	5 × 10^5^	3 mm diameter3 mm depth	Press-fit
Yoshioka T, et al.	J Tissue Eng Regen Med	2011	Rabbit	Rotatory Culture(Bioreactor)	Spheroid	Bone MarrowMSC	Mainly hyaline-like appearance	1.5 - 3.0 × 10^7^	5 mm diameter4 mm depth	Nether glue nor suture
Qu C, et al.	Cell Tissue Res	2012	in vitro	Transwell Culture	Disc	Chondrocyte	Catilage-like	6 × 10^6^	N/A	N/A
^a^Ando W, et al.	Eur Cell Mater	2012	Pig	High-density in Monolayer	Sheet	Synovial MSC	Collagen I, III-rich	4 × 10^5^/cm^2^per plate	8.5 mm diameter2 mm depth	Nether glue nor suture
Ebihara G, et al.	Biomaterials	2012	Minipig	Layered Chondrocyte Sheet	Sheet	Chondrocyte	Catilage-like	5 × 10^4^per cm^2^	6 mm diameter5 mm depth	Suture
Sato Y, et al.	J Biosci Bioeng	2013	in vitro	Culture on Membrane (polyethylene terephthalate)	Disc	Bone MarrowMSC	Catilage-like	1.86 × 10^6^	N/A	N/A
Brenner JM, et al.	Biotechnol Prog	2013	in vitro	Bioreactor	Sheet	Chondrocyte	Catilage-like	2 × 10^4^	N/A	N/A
Giardini-Rosa R, et al.	Tissue Eng Part A	2014	in vitro	Bioreactor	Sheet	Chondrocyte	Catilage-like	6.5 or 13 × 10^3^per cm^2^	N/A	N/A
Mohanraj B, et al.	J Biomech	2014	in vitro	High-density in Monolayer	Disc	Chondrocyte	Catilage-like	1 × 10^6^	N/A	N/A
Liu T, et al.	Tissue Eng Part A	2014	in vitro	Self Assembling	Disc	Fat Pad-derivedStem Cell	Catilage-like	3.8 × 10^6^	N/A	N/A
Ylärinne JH, et al.	Cell Tissue Res	2014	in vitro	Transwell Culture	Disc	Chondrocyte	Catilage-like	6 × 10^6^	N/A	N/A
Oda K, et al.	J Orthop Sci	2014	Rabbit	High-density in Monolayer	Disc	Chondrocyte	Catilage-like	6 × 10^6^per cm^2^	4 mm diameter(Osteochondral defect)	Not addressed
Ishihara K, et al.	J Orthop Surg Res	2014	Rabbit	Spheroid Formation	Cylinder	Bone MarrowMSC	Not stained with Safranion O	4 × 10^4^per well	4.8 mm diameter4-5 mm depth	Nether glue nor suture
Brenner JM, et al.	Artificial Organs	2014	Rabbit	Bioreactor	Sheet	Chondrocyte	Catilage-like	2 × 10^4^	4 mm diameter(Chondral only defect)	Fibrin glueand Suture
^a^Fujie H, et al.	J Biomech	2015	Pig	High-density in Monolayer	Sheet	Synovial MSC	Collagen I, III-rich	4 × 10^5^/cm^2^per plate	8.5 mm diameter2 mm depth	Nether glue nor suture
Yamashita A, et al.	Stem Cell Reports	2015	Minipig	Staged Differentiation Media	Spheroid	iPSC	Catilage-like	1-2 × 10^5^	Not addressed	Fibrin glue
He P, et al.	Acta Biomater	2016	in vitro	Culture on Alginate-based Micro-cavity Hydrogel	Disc	iPSC	Catilage-like	1 × 10^7^per mL	N/A	N/A
Islam A, et al.	Eur Cell Mater	2016	in vitro	Pellet Culture	Spheroid	Umbilical Cord MSC	Catilage-like	5 × 10^4^	N/A	N/A
^a^Yasui Y, et al.	Tissue Eng Part A	2016	in vitro	High-density in Monolayer	Sheet	Synovial MSC	Catilage-like	4 × 10^5^/cm^2^per plate	N/A	N/A
Kimura T, et al.	Tissue Eng Part A	2016	in vitro	Staged Differentiation Media	Spheroid	iPSC	Catilage-like	1-2 × 10^5^	N/A	N/A
Bartz C, et all.	J Transl Med	2016	ex vivo	Self Aggregating	Spheroid	Chondrocyte	Catilage-like	2 × 10^5^	4 mm diameter	Nether glue nor suture
^a^Koizumi K, et al.	OsteoarthritisCartilage	2016	Rat	High-density in Monolayer	Sheet	Synovial MSC	Collagen I, III-rich	4 × 10^5^/cm^2^per plate	1.5 mm diameter1 mm depth	Nether glue nor suture
Itokazu M, et al.	Cartilage	2016	Rat	Culture on Membrane (polyethylene terephthalate)	Disc	Bone MarrowMSC	Catilage-like	1.86 × 10^6^	2 mm diameter1 mm depth	Fibrin glue
Becher C, et al.	J Orthop Surg Res	2017	Clinical	Self Aggregating	Spheroid	Chondrocyte	Catilage-like	2 × 105	4 - 10 cm2ICRS grade III or IV	Not addressed

*MSC* mesencymal stem cell, *PRP* platelet rich plasma, *iPSC* induced pluripotent stem cell

^a^:Our study

To date, there have been several scaffold-free techniques developed. DuRaine et al. defined such techniques to divide two categories, self-organization and self-assembly, according to the fabrication method (DuRaine et al. 2015). Self-organization describes a process in which order appears when external energy or forces are input into the system, including bioprinting and cell-sheet engineering. Also, cell aggregates are commonly formed in culture by applying a rotational force to cells in suspension or other non-adherent culture conditions, and thus that is categorized as a self-organization. On the other hand, a self-assembling process is a tissue engineering technique that does not employ external forces to form tissues. The process of tissue maturation follows a course similar to that of native cartilage development, in which cellular interactions and coalescence (e.g. high-density cell culture) are driven by spontaneous minimization of free energy, and then tissue-specific extracellular matrix (ECM) is produced to form functional tissue via maturation process.

Regarding cell selection in a scaffold-free approach, chondrocytes have been mostly employed to generate neocartilage (Table 1). These cells readily produce their tissue-specific ECM, especially in 3D culture environment (Huang et al. 2016). On the other hand, the limited cell availability and dedifferentiation potential during cell expansion might be a concern as mentioned above, although chondrocyte-based neocartilage is reported to achieve biochemical and biomechanical values within the range for native cartilage (Mainil-Varlet et al. 2001; Mohanraj et al. 2014; Ebihara et al. 2012; Jubel et al. 2008). In addition, the implantation of such a neocartilage usually needs to be fixed with fibrin glue, suture, or a periosteal covering, since these tissues might not exhibit adhesive properties required to integrate with host cartilage (Brehm et al. 2006; Jubel et al. 2008; Lewis et al. 2009; Ebihara et al. 2012). As an alternative, MSCs and iPS cells have been recently tested (Table 1), and an engineered tissue generated from these cells showed feasibility for cartilage repair comparable to chondrocyte-based tissues (Murdoch et al. 2007; Ishihara et al. 2014; Yamashita et al. 2015).

Interestingly, some research has progressed to the stage of preclinical studies using a large animal model and clinical study, and we introduce such recent scaffold-free approaches with high potential clinical impact. Mainil-Varlet et al. developed a cartilage-like implant in chondrocyte high density culture supported by a bioreactor, and implanted the materials onto minipig cartilage defects by press-fit fixation (Mainil-Varlet et al. 2001). Histological analysis showed such an implant yielded consistent cartilage repair with a matrix predominantly composed of type II collagen. Lu et al. produced a neocartilage allograft under defined serum-free conditions, and transplanted such graft, which were cultured to produce these constructs between 107 and 130 days, onto sheep chondral defects with suturing (Lu et al. 2005). Eight-week histology showed the reparative tissue appeared to be hyaline-like with weak Safranin O staining, and no inflammatory cells were observed around the grafted area. Park et al. produced chondrocyte/ECM membranes in high density culture, peeled the membrane from the culture dishes, consolidated them by centrifuge, and then generated an engineered cartilage construct by an additional culture period (Park et al. 2006). Jubel et al. reported that chondrocytes cultured in alginate beads for 21 days, then collected by centrifuge after the beads were dissolved, and then additionally cultured for 7 days in a cylinder mold formed cartilage-like de novo tissue (Jubel et al. 2008). These two authors further demonstrated the feasibility of their cartilage-like constructs for cartilage repair in vivo, but the implanted constructs needed to be fixed with collagen gel and periosteal flap, respectively. Ebihara et al. used layered chondrocyte sheets prepared on a temperature-responsive culture dish, and demonstrated these constructs facilitated cartilage repair in a minipig model (Ebihara et al. 2012). With this technique, cultured cells could be harvested noninvasively from the dishes by reducing only temperature (Kushida et al. 2000). Moreover, since the harvest did not need enzymatic digestion, differentiated cell phenotypes were retained.

As another scaffold-free approach, chondrocytes were cultured at high density by cell aggregation to fabricate engineered cartilage constructs (Huang et al. 2016). Cells were aggregated, adhered each other as spheroids in a few hours, and, after an additional few weeks culture, cartilage-specific matrices were secreted to subsequently form solid neotissues (Anderer and Libera 2002). Such a neotissue contributed to the regeneration of full thickness cartilage defects in a pig study (Libera et al. 2009). Additionally, Becher et al. treated 73 patients with chondral lesion (ICRS grade III or IV) using these neotissues, and showed well tolerated clinical results without any serious adverse events (Becher et al. 2017). On the other hand, with this method, the size of the tissue was limited due to its diffusion limit (DuRaine et al. 2015), and thus a large number of small aggregates may be required to cover a large cartilage defect.

More recently, Yamashita et al. developed a scaffold-less hyaline cartilaginous tissue (particle) from human iPS cells (Yamashita et al. 2015). These iPS cell-derived cartilaginous particles were feasible for use in hyaline cartilage regeneration based on the results of a mini-pig study, although these constructs required fixation with fibrin glue due to their non-adhesive properties.

In summary, many promising scaffold-free approaches have been developed until now, and such technologies could become a next generation vehicle for cartilage repair, with regard to a high level of safety by avoiding extrinsic materials (Huey et al. 2012). On the other hand, there are still several issues that need to be solved prior to future clinical applications such as complicated fabrication methods, long culture periods, and the nonadhesive properties of generated tissues needed to be overcome for integration with host cartilage. Moreover, a large number of cells may be required to cover large chondral lesions since a scaffold-free approach lacks exogenous materials and must promote ECM production by the cells themselves.

To address several of these issues mentioned above, we have developed a novel scaffold-free 3D tissue engineered construct (TEC) that is comprised of either human or porcine MSCs derived from synovium and an ECMs synthesized by the cells (Shimomura et al. 2015). The safety and effectiveness of this TEC methodology for cartilage repair and regeneration will be focused on hereafter, as it moves from bench to bedside for clinical application.

### In vitro development of a scaffold-free tissue engineered construct (TEC) derived from MSCs

**Fig. 1:**
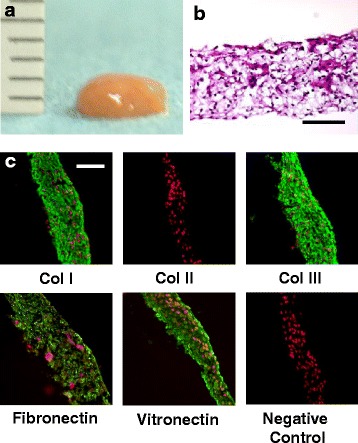
Development of the tissue-engineered construct (TEC). **a** Macroscopic view of the TEC that was integrated to one spherical body. **b** Hematoxylin and eosin staining of TEC. **c** Immunohistochemical analysis of the TEC stained with type I collagen (Col I), type II collagen (Col II), type III collagen (Col III), fibronectin, vitronectin, and negative IgG (control). Red are nuclei and green is target antibody. Adhesion molecules such as fibronectin and vitronectin are diffusely distributed within the TEC. Bar = 100 μm. Quoted and modified from *Ando* et al.*, Biomaterials 2007 and Shimomura* et al. *Cartilage 2015*

Synovial membrane harvested from either porcine or human knee joints was enzymatically digested, and synovial MSCs were isolated, and then expanded in growth media containing virus- and prion-free fetal bovine serum. The isolated cells showed characteristics of MSCs with regard to morphology, growth characteristics, and multipotent differentiation capacity (to osteogenic, chondrogenic, and adipogenic lineages) (Ando et al. 2007; Ando et al. 2008). When synovial MSCs were cultured to confluence in a basic growth medium, they did not synthesize an abundant collagenous matrix. However, in the presence of >0.1 mM ascorbic acid-2 phosphate (Asc-2P), collagen synthesis significantly increased with time in culture (Ando et al. 2008). Subsequently, the monolayer cell-matrix complex cultured in Asc-2P became a stiff sheet-like structure. After detachment from culture dishes by mild shear stress, the monolayer sheet immediately began to actively contract and form a thick 3D tissue (Fig. 1a). Histology of this 3D tissue showed that the cells and the corresponding ECM were three dimensionally integrated together at high cell density without evidence for the appearance of central necrosis (Fig. 1b). Immunohistochemical analysis showed that such TEC were rich in collagen I and III, but with no detectable expression of collagen II (Fig. 1c). Interestingly, adhesive molecules such as fibronectin and vitronectin were diffusely observed throughout the matrix of such TEC **(**Fig. 1c**)**. These characteristics likely contribute to the expression of the highly adhesive properties of the TEC. As the TEC develops when the matrix becomes folded and contracted, it was thus apparent that the layers were integrated into each other, leading to development of a spherical body several millimeters thick. The contraction feature of the TEC is likely due to the presence of alpha-smooth muscle actin positive cells (Ando et al. 2008). The TEC generated were of a sufficient size to cover a large cartilage defect. This contracted tissue was termed a tissue engineered construct (TEC) derived from MSCs.

### Chondrogenic differentiation capacity of the TEC

**Fig. 2:**
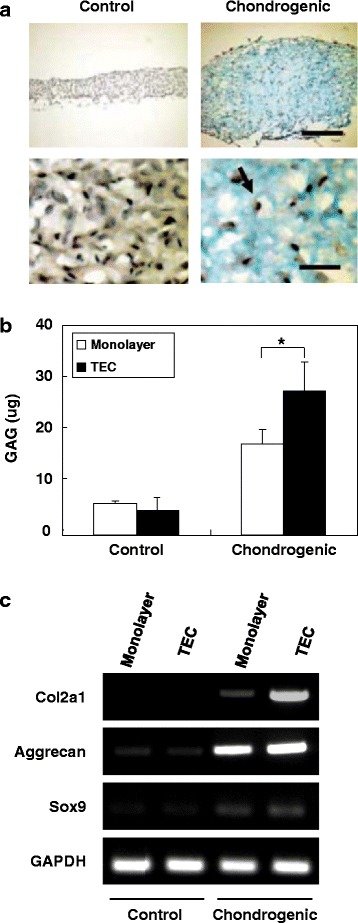
Chondrogenic differentiation capacity of TECs. **a** Alcian blue staining of a TEC in control medium or in chondrogenic differentiation medium. Bar **=** 300 um (upper). Bar **=** 50 um (lower). Arrow showing the cell nuclei are in lacuna. **b** The quantification of GAG content of synovial MSC monolayer culture or TEC in the control medium or in the chondrogenic medium. GAG synthesis is significantly higher in the TEC exposed to the chondrogenic medium (*N* = 8). *; *p* < 0.05. **c** Semiquantitative reverse transcription–polymerase chain reaction (RT-PCR) analysis of synovial MSC monolayer culture or TEC for chondrogenic marker genes, type II collagen (Col2a1), aggrecan, SOX9, and GAPDH. Quoted and modified from *Ando* et al.*, Tissue Eng Part A 2008*

TEC derived from human MSCs and then cultured in a chondrogenic medium containing BMP-2 showed increased glycosaminoglycan (GAG) synthesis and deposition as evidenced by intense Alcian Blue staining (Fig. 2a). The quantification of GAGs indicated that GAG synthesis was significantly higher in the TEC exposed to the chondrogenic medium compared to those generated in the absence of such components (Fig. 2b). Similarly, semiquantitative RT-PCR analyses showed elevations in expression of cartilage-specific markers including collagen II (Col2a1), aggrecan, and sox9 messenger RNA (mRNA) following exposure of the TEC to the chondrogenic differentiation medium (Fig. 2c). In contrast, TEC not exposed to this medium showed only weak expression of these cartilage-specific markers.

### Selection of a preclinical large animal model

One of the crucial factors that may affect the results of cell-based therapies is the age of the donors and the recipients. Regarding the cell proliferation and differentiation capacities of MSCs, it is controversial as to whether they are age-dependent (Murphy et al. 2002; Quarto et al. 1995; Bergman et al. 1996; Kretlow et al. 2008) or not (De Bari et al. 2001; Oreffo et al. 1998; Leskela et al. 2003; De Bari et al. 2001; Scharstuhl et al. 2007). In terms of the host tissue reaction, natural healing responses of osteochondral defects has been compared between immature and mature animals using rabbit models, and in this species, the studies demonstrated better healing responses in immature animals (Rudert 2002; Bos et al. 2006; Yamamoto et al. 2004; Wei et al. 1997). On the other hand, there have been no studies reported which directly compared the results of cell-based repair of chondral defects between immature and mature animal models.

Regarding the use of a clinically relevant animal model for cartilage repair, it is difficult to create a chondral injury which does not breach the subchondral bone in small animals such as rabbits, rats, and mice due to the limited thickness of their articular cartilage, and therefore, these conditions may not be as clinically relevant as would be obtained with the use of a larger animal. Thus, in consideration of clinical relevance, it is preferable to utilize a large animal model to investigate the influence of maturity on the results of cell-based therapies to repair chondral lesions. Therefore, in order to assess the efficacy of the TEC in an in vivo model, a porcine model was chosen as the physiology of the pig is similar to that of humans in many respects (Vodicka et al. 2005), and porcine articular cartilage of the knee is sufficiently thick as to allow creation of a chondral defect without damaging the subchondral bone.

### Cartilage repair using TECs in a preclinical large animal study

Prior to performing a large animal study, we compared the in vitro characteristics of cell proliferation and chondrogenic capacity of porcine MSCs isolated from skeletally immature animals (3–4 month old) with those from mature animals (12 month old). We demonstrated that there were no significant differences in either the proliferation or chondrogenic capacity of porcine synovial MSCs derived from immature or mature animals (Shimomura et al. 2010).

**Fig. 3:**
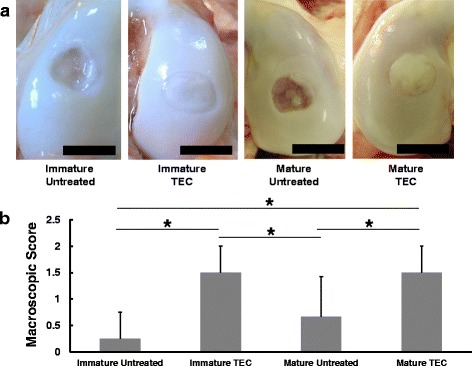
**a** Macroscopic view of a chondral lesion treated with or without TEC at 6 months post-implantation. Bar = 10 mm. **b** Macroscopic score of the repair tissue at 6 months. Regardless of skeletal maturity, the TEC group shows significantly higher scores than does the untreated group. *; *p* < 0.05. Quoted and modified from *Shimomura* et al.*, Biomaterials 2010*

**Fig. 4:**
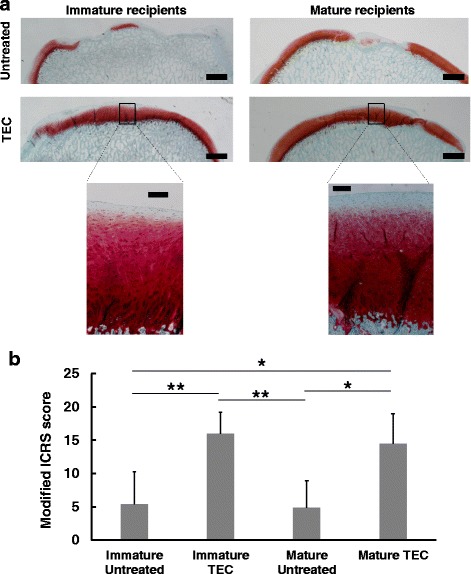
**a** Safranin O staining of chondral lesions treated with or without TEC. Bar = 1 mm (upper). Bar = 200 um (lower). Regardless of skeletal maturity, the defects treated with TEC are completely filled with Safranin O positive repair tissue with good tissue integration. **b** Modified ICRS score for repair cartilage in skeletally immature and mature recipients. The TEC group exhibits significantly higher scores than does the untreated control group, regardless of maturity. *; *p* < 0.05, **; *p* < 0.01. Quoted and modified from *Shimomura* et al.*, Biomaterials 2010*

To test the feasibility of using the porcine TEC approach for a wide range of recipient ages without chondrogenic manipulation to repair a chondral injury, immature (TEC implantation for eight knees and untreated control for four knees) as well as mature (TEC implantation for six knees and untreated control for six knees) porcine chondral injury models were utilized in experimental studies. After implantation, the TEC firmly adhered to the injured joint surface without suturing or any glues. At 6 months post-implantation, and regardless of starting age, untreated lesions exhibited no evidence for repair or only partial tissue coverage, while the defects treated with a TEC were totally or primarily covered with repair tissue (Fig. 3a, b). Histologically, the chondral lesions in the non-treatment control groups showed evidence of osteoarthritic changes, with loss of cartilage and destruction of subchondral bone in both skeletally immature and mature animals (Fig. 4a). In contrast, when treated with a TEC, the defects were filled with repair tissue exhibiting good integration to the adjacent cartilage and restoration of a smooth surface, regardless of age at the time of implantation (Fig. 4a). The repair tissue exhibited predominantly spindle-shaped fibroblast-like cells in the superficial zone of the repair tissue, while the majority of the remaining repair matrix contained round-shaped cells in lacuna (Fig. 4a). Following implantation, no histological findings were obtained that suggested either central necrosis of the implanted TEC, or that an abnormal inflammatory macrophage and lymphocyte response consistent with some form of immunological rejection had occurred in this allogenic situation, regardless of the age of the pigs. Histological scoring showed that the TEC groups exhibited significantly higher scores than did the untreated control group, regardless of skeletal maturity (Fig. 4b). Comparing the repair tissues developing following TEC implantation in immature and mature animals, no significant detectable differences were detected at the histological level (Fig. 4b).

**Fig. 5:**
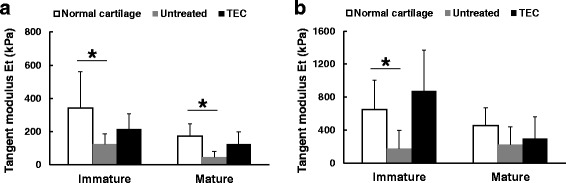
The results of compression tests at a slow compression speed (4 um/s) (**a**) and at a fast compression speed (100 um/s) (**b**). Regardless of skeletal maturity, no significant differences were detected in the tangent modulus of the TEC-mediated repair tissue and that of normal cartilage at either compression speed. Conversely, untreated defects, whether in immature or mature recipients, showed significantly lower tangent modulus than did normal cartilage at either compression speed. *; *p* < 0.05. Quoted and modified from *Shimomura* et al.*, Biomaterials 2010*

To assess the viscoelasticity of the repair cartilage (Huang et al. 2003), we performed in vitro compression testing at both fast and slow speeds. In the tissue localized to the defects of the untreated control group, the tangent modulus (defined as the slope of the curve at 5% strain) was significantly lower than that for normal cartilage at a compression rate of either 4 μm/s (Fig. 5a) or 100 μm/s (Fig. 5b), regardless of skeletal maturity of the pigs. In contrast, there were no significant differences detected between the tangent modulus for the repair tissue resulting from implantation of a TEC and that for normal cartilage at either 4 μm/s (Fig. 5a) or 100 μm/s (Fig. 5b) in either the immature or mature animals. These results suggest that the viscoelastic properties of the repair tissue in defects receiving TEC implants are likely very similar to those of normal cartilage, regardless of host skeletal maturity at the time of implantation.

Based on the encouraging results of the preclinical studies discussed above, we have now proceeded to clinical studies under the auspices of an approved first in man protocol by the institutional review board of Osaka University Graduate School of Medicine (Nakamura et al. 2014). This pilot clinical study is currently in progress, and the data are now under analysis (Shimomura et al. 2015).

### Implications of the findings from the preclinical studies on TEC-use for cartilage repair in vivo

Based on the findings to date, the exogenous scaffold-free TEC approach offers a number of advantages for hyaline cartilage repair. Firstly, since the TEC develops without any exogenous scaffold, implantation of the TEC would likely have minimal risk of potential side effects induced by artificial or extrinsically added biological materials contained in a scaffold. On the other hand, the MSCs were exposed to certified virus- and prion-free fetal bovine serum (FBS) during cell culture in the present studies. Thus, there are some concerns regarding this exposure of the TECs to FBS-origin proteins during the development process. Although the TEC were washed extensively in vitro with sterile phosphate buffered saline, it cannot be concluded that no FBS proteins were retained in the constructs. To address this issue, we have confirmed that human serum is no less effective than bovine serum in promoting proliferation of synovium-derived MSCs and their chondrogenic capacities (Tateishi et al. 2008). Accordingly, with the use of autologous human serum, it is technically possible to develop the TEC in a totally xeno-free system for cartilage repair, a set of circumstances which would minimize the risk of infectious agents, as well as potential immune reactivity developing after implantation of the TEC (Martin et al. 2005).

Secondly, a further structural advantage of the TEC is that the MSCs and the ECM synthesized by the cells are integrated together into a 3D structure with a uniform cellular distribution. Thus, there is no need to modify or adjust the cellular distribution within the TEC. It is also notable that these TEC possess sufficiently self-supporting mechanical properties in spite of the fact that they do not contain an exogenous artificial scaffold. The tensile strength of the TEC, which develops in the presence of Asc-2P for 14 or 21 days, is comparable with that of healing ligament tissue at 1–2 weeks after injury (Provenzano et al. 2002). Therefore, such TEC can be readily handled without causing overt damage to the matrix-cell complex during implantation procedures.

Thirdly, an important biological characteristic of the TEC described is its tissue adhesiveness. This property contributes to the rapid and secure adhesion of the TEC to a natural cartilage matrix and thus, simple implantation procedures for the placement of the TEC into chondral lesions or defects could be expected to proceed without augmentation of the initial fixation. Moreover, such adhesiveness also enables rapid self-association internally with its own matrix, a factor which likely contributes to the tissue plasticity of the TEC. In reality, it is thus possible to develop a spherical-shaped tissue several millimeter thick by allowing the released monolayers from several dishes to fold in series. With such “plasticity”, it is possible to develop a TEC that matches the needed size and shape to repair a chondral defect more than several millimeters in depth. Although we have not yet identified the crucial factor(s) which determine the tissue adhesiveness of the TEC, immunohistochemical analysis has shown that fibronectin and vitronectin are localized at the interface between the TEC and the base of the chondral lesions. Therefore, fibronectin and vitronectin may likely be, at least partially, involved in the adhesive properties of these in vitro generated TEC.

Fourthly, it is notable that implantation of TEC without any pretreatment to promote a specific differentiation pathway resulted in tissue repair associated with an active chondrogenic differentiation response. Thus, the implantation of a basic TEC lead to the in vivo differentiation of such TEC in response to the in vivo biological and biomechanical cues following differentiation. Therefore, in vitro differentiation may not offer additional value to the success of the repair. On the other hand, it is still controversial whether implanted MSCs directly contribute to cartilage repair and are retained within the hyaline-like tissue that develops post-implantation, or they interact with the surrounding environment through the release of anti-inflammatory and trophic mediators and facilitate involvement of endogenous cells (Caplan and Hariri 2015). Therefore, further studies are required to determine whether the implanted TEC contributed to cartilage repair directly by local differentiation to chondrocytes or indirectly via release of mediators which enhance repair by activation of endogenous chondrocytes or activation and differentiation of other cells in the intra-articular environment.

However, based on the outcomes from the porcine preclinical studies, the in vivo repair was not perfect, in that the repair tissue still contained some fibrous tissue, mainly at the surface or in the superficial zone. In the detailed biomechanical studies discussed above, the TEC-mediated repair cartilage still exhibited some compromised mechanical properties at the upper level of the superficial zone (lamina splendens), a deficiency that will likely need improvement in the future for maintenance of long term repair cartilage integrity (Ando et al. 2012). However, as the lamina splendens apparently develops during the post-natal period in some species (Takada et al. 1999; Fujioka et al. 2013; Gannon et al. 2015), further understanding of this process could provide a solution to the observed structural deficiency following in vivo TEC implantation.

### Future perspectives for the scaffold-free MSC-based TEC approach to repair cartilage

Building on the results of the preclinical studies, the present review has provided the current evidence for the safety and feasibility of using a unique scaffold-free TEC generated from synovial MSCs for effective cell-based cartilage repair in a clinical setting. Additionally, this technique is simple and should be easy for surgeons to handle for implantation into cartilage defects. Thus, such a new MSC-based technique could be considered as the next generation vehicle for cartilage repair.

Cartilage injuries might become curable with currently available cell-based therapies. On the other hand, the bigger clinical problem is related to the higher incidence of osteoarthritis (OA), an incidence that is much higher than that for isolated chondral injuries (Hjelle et al. 2002; Aroen et al. 2004; Dawson et al. 2004; Peat et al. 2001). Therefore, development of novel therapeutic methods for osteochondral repair are also urgently needed, considering the large population of patients with early and advanced osteoarthritis. As such lesions very often involve subchondral bone damage, it is important to also consider subchondral bone regeneration in addition to cartilage. Recently, we have combined the scaffold-free MSC-based TECs with an artificial bone block to fabricate a biphasic osteochondral implant, and demonstrated the feasibility of using such constructs for osteochondral repair in a rabbit study (Shimomura et al. 2014; Shimomura et al. 2017). Therefore, the combined TEC-artificial bone construct as another viable option for TEC application, could also be considered a promising MSC-based bio-implant to repair osteochondral lesions in the near future.

## Conclusion

In this review, we have focused on recent advancements in scaffold-free approaches for cartilage repair. Many promising techniques have been developed and some of them have demonstrated their feasibility to repair cartilage lesions in preclinical and clinical studies. Therefore, the clinical application of such new technologies could be expected in near future. On the other hand, the optimization of and selection of cell sources and their fabrication methods have not been fully investigated, without any direct comparative studies. Thus, the ideal scaffold-free approaches that lead to repair of cartilage lesions have not been elucidated in detail. Further studies are needed and should be conducted in a methodologically rigorous fashion.

Notably, we have elucidated many of the characteristics of a scaffold-free 3D synthetic tissue (TEC) derived from cultured synovium-derived MSCs as a unique and promising clinically relevant implant for cartilage repair. This was demonstrated in vivo using a preclinical model and a range of ages (Shimomura et al. 2010; Ando et al. 2007; Ando et al. 2008). Due to the scaffold-free nature of their in vitro generated structure, implantation of TEC could potentially yield more long-term safety and efficacy than other options derived from scaffold-based cell therapies. Being a collagen I rich matrix, the basic TEC construct could also be potentially suitable for augmenting repair of compromised skin, or enhancing the repair of ligaments or tendons, which are also collagen I rich. Since TEC also have osteogenic and adipogenic differentiation capacity (data not shown) in addition to chondrogenic potential, basic TEC could likely also be used for other applications. Moreover, TEC could also be developed from MSCs derived from other tissues, such as adipose tissue which is an abundant source of MSC and readily obtained without entering the damaged joint. Therefore, tissue engineering using the TEC technology discussed could potentially provide a variety of therapeutic interventions for regenerative medicine in a number of tissue applications using MSC from different sources. However, as MSC populations from a given source are very heterogeneous, and such heterogeneity can vary between tissue sources (Ando et al. 2014; Hart 2014), this potential of the TEC technology will have to be rigorously characterized. Whether clonally-derived MSC versus specific MSC populations will be more efficacious for particular applications, remains to be determined.

## Abbreviations

3D: Three dimensional
ACI: Autologous chondrocyte implantation
Asc-2P: Ascorbic acid-2 phosphate
ECM: Extracellular matrix
GAG: Glycosaminoglycan
iPS cells: Induced pluripotent stem cells
KOOS: Knee injury and osteoarthritis outcome score
mRNA: messenger RNA
MSC: Mesenchymal stem cell
RT-PCR: Reverse transcription–polymerase chain reaction
TEC: Tissue engineered construct
